# Feather Damage Monitoring System Using RGB-Depth-Thermal Model for Chickens

**DOI:** 10.3390/ani13010126

**Published:** 2022-12-28

**Authors:** Xiaomin Zhang, Yanning Zhang, Jinfeng Geng, Jinming Pan, Xinyao Huang, Xiuqin Rao

**Affiliations:** 1 College of Biosystems Engineering and Food Science, Zhejiang University, 866 Yuhangtang Road, Hangzhou 310058, China; 2 Key Laboratory of Intelligent Equipment and Robotics for Agriculture of Zhejiang Province, Hangzhou 310058, China; 3 School of Mechanical and Electrical Engineering, Zaozhuang University, Beian Road, Zaozhuang 277160, China

**Keywords:** poultry, feather damage monitoring, 3D reconstruction, RGB-D, infrared thermography, deep learning

## Abstract

**Simple Summary:**

Feather coverage reflects the production efficiency and animal welfare of poultry. Monitoring the feather-cover condition of chickens is of great significance. Infrared thermography can be used to evaluate the probable existence of inflammatory or tissue damage processes due to the variation in skin temperature, which can be used to objectively determine the depth of the feather damage. In this study, a 3D reconstruction pipeline of chicken monitoring was developed, with color, depth and thermal information for the comprehensive feather damage monitoring of chickens. The results demonstrated that the proposed method can better assess the feather damage compared to a 2D color image or thermal infrared image. The depth of chicken feather damage can be assessed by the 3D model. The method provided ideas for automation and intelligent feather-damage monitoring in poultry farming.

**Abstract:**

Feather damage is a continuous health and welfare challenge among laying hens. Infrared thermography is a tool that can evaluate the changes in the surface temperature, derived from an inflammatory process that would make it possible to objectively determine the depth of the damage to the dermis. Therefore, the objective of this article was to develop an approach to feather damage assessment based on visible light and infrared thermography. Fusing information obtained from these two bands can highlight their strengths, which is more evident in the assessment of feather damage. A novel pipeline was proposed to reconstruct the RGB-Depth-Thermal maps of the chicken using binocular color cameras and a thermal infrared camera. The process of stereo matching based on binocular color images allowed for a depth image to be obtained. Then, a heterogeneous image registration method was presented to achieve image alignment between thermal infrared and color images so that the thermal infrared image was also aligned with the depth image. The chicken image was segmented from the background using a deep learning-based network based on the color and depth images. Four kinds of images, namely, color, depth, thermal and mask, were utilized as inputs to reconstruct the 3D model of a chicken with RGB-Depth-Thermal maps. The depth of feather damage can be better assessed with the proposed model compared to the 2D thermal infrared image or color image during both day and night, which provided a reference for further research in poultry farming.

## 1. Introduction

Poultry welfare influences the performance and disease rate of poultry flocks [1]. Feather cover condition is an important indicator of poultry welfare [2]. Intact feather cover ensures normal moving, flying, thermoregulation and skin protection for the chickens [3,4]. However, poultry may lose their feathers due to feather pecking in flocks [4] or abrasion and molting [5]. Feather pecking is closely associated with feather loss and skin injuries, particularly on the rump and back [6,7]. Experimental studies have identified several factors influencing feather pecking, such as genotype, nutritional factors, group size and stocking density, light, the frequency of resting and dust bathing, flooring (wire or substrate and type of litter), fear and stress. [8,9,10,11]. When chickens cannot perch inside their cages [12] or the substrate does not permit dust bathing in hens, chickens are more likely to perform severe feather pecking [13]. Therefore, convective heat loss from poorly feathered chickens is increased. This leads to an increase in heat loss and feed consumption, and a decrease in egg production [14], which may cause economic losses for the farmer [15]. The causes of feather pecking are mainly associated with the lack of enrichment [16], high and low feather-pecking lines [17], which are sometimes not taken into account in production units. Therefore, it is important to develop objective and practical tools that can evaluate feather damage.

The commonly utilized method for feather-cover assessment was traditional feather scoring (FS) [18], which was a visual evaluation of the feather on different parts of the body. However, this method is relatively subjective and less repeatable [19]. Feathers act as thermal insulation and affect the heat exchange between the hen’s body and the surroundings [20]. A hen with poor feather coverage loses more body heat compared to that with good feather coverage, therefore, requires thermoregulation [21]. Infrared thermography (IRT) detects the heat radiation emitted from the surface of an organism. [22] IRT can quantify and evaluate surface temperatures in the external skin of animals, which are dependent on blood circulation [23]. As circulation increases, it is possible to observe the condition of the internal organs using IRT. This allows for an evaluation of the probable existence of inflammatory or tissue damage processes due to the variation in skin temperature [24]. The featherless region, with heat loss in the infrared spectrum, can be detected by IRT, manifested as different thermal images [25]. IRT has been increasingly applied to monitor the physiological and health status of animals [26]. The acquisition of hen surface temperature by the IRT allows for a feather damage assessment, which is more objective, accurate and repeatable [27]. Cook et al. [19] compared a feather cover assessment of laying hens using IRT with the feather scoring method. The IRT method was confirmed to provide a more objective and accurate measurement with continuous variables. However, the defined temperature ranges for the featherless area were subjective. Nääs et al. [28] reported a significant relationship between the radiated temperature and feather cover. They found that featherless areas are more sensitive to air temperature than feathered areas. Zhao et al. [23] showed that the IRT is a promising tool compared with FS. Based on the 2D thermal infrared images, the body surface temperature ranges were analyzed to discern excellent feather, fair feather, and no feather coverage of different body parts of laying hens. Pichová et al. [29] utilized IRT to assess feather damage in four body parts. The difference between body surface temperature and ambient temperature was positively correlated with feather score. However, the assessment process was non-automatic.

Recently, many scholars have conducted health monitoring and inflammation evaluations of poultry using machine vision technology [30,31,32,33]. Nevertheless, these studies are mainly based on 2D RGB or thermal infrared images. Feather damage assessments could not assess the depth of feather damage or comprehensively evaluate the extent of the feather damage. Tissue pecking in denuded areas involves forceful pecking directed at exposed skin and may cause bleeding, which may attract even more pecking with other birds joining in, leading to severe skin damage and possibly death [4]. It is crucial to assess the depth of feather damage. To more accurately and automatically monitor the feather damage condition of chickens, an effective and automated multi-data fusion model including RGB, depth and thermal information would be a better solution. RGB images are provided with a rich texture and color features with high resolution. Thermal imaging allows for detection in a low illumination at night. The fusing information obtained from these two bands can highlight their strengths, which is more evident in the assessment of feather damage. Additionally, if the fused images contain depth information, the depth of feather damage can be determined.

Generally, the 3D data can be obtained by binocular, time-of-flight (TOF), structured light cameras or light detection and ranging (LiDAR) [34]. The current techniques to reconstruct the 3D model with multi-source information rely on merging data from different sensors. These techniques can be divided into two categories: (i) The first method is to map the RGB and thermal information to a 3D point cloud. Susperregi et al. [35] presented a multi-sensor fusion approach that combined an RGB-D camera, a laser and a thermal sensor on a mobile platform for pedestrian detection and tracking. In addition to RGB-D cameras, LiDAR was also used for depth data acquisition [36]. Subsequently, some scholars also studied RGB-Depth-Thermal (RGB-D-T) reconstruction in human medicine. A multimodal sensor system including RGB, 3D depth, thermal, multispectral and chemical sensing was presented for wound assessment and pressure ulcer care [37]. An infrared thermal camera was combined with an RGB-D sensor to necrotize enterocolitis detection in preterm human infants [38]. Although depth cameras and range sensors could provide accurate point clouds, their precision and range were still affected during operation outdoors. (ii) The second method is an image-based point cloud reconstruction. Lagüela et al. [39] proposed a semi-automatic pipeline to generate an RGB-D-T model by image-stitching and surface reconstruction. An operator is still needed to verify the matches between RGB and thermal images. Ham and Golparvar-Fard [40] solved the image registration problem (the process of transforming different sets of data into one coordinate system to achieve image alignment) by simultaneously capturing RGB and thermal images. However, the multi-view stereo process [41] used for 3D reconstruction would take much more time. Several RGB-thermal point cloud registration approaches were assessed by Hoegner et al. [42]. The most accurate proposed method was based on the Iterative Closest Point (ICP) algorithm [43]. Generally, the key to the image-based point cloud reconstruction is to align the RGB and thermal images.

Although much progress was achieved in previous studies on 3D reconstruction with multi-source information, the following problems need improvements: (i) Traditional stereo matching algorithms suffered in challenging situations, such as in weak texture regions and repetitive patterns, which led to considerable errors in estimated depth data [44]. (ii) Typically, an RGB image is of high resolution and has rich texture information, while the thermal infrared image is of low resolution and has poor texture details. Traditional feature-based image registration methods [45] calculate the optimal homography matrix by establishing a correspondence between the two sets of key points [46]. However, the accuracy of these methods depends on the numbers of detected key points and the correct key points correspondences, which may be difficult to apply and cannot guarantee the fusion of heterogeneous images. (iii) The segmentation process of the RGB images for chickens is important for the accuracy of the reconstruction. The fully convolutional networks (FCN) [47], U-Net [48] and other fully convolutional-based networks, such as SegNet [49] and DeepLab [50], have shown great potential in semantic segmentation tasks. Nevertheless, the chicken’s claws are very thin and the feathers are easily confused with the surrounding background, which increases the complexity of the task.

To overcome these limitations, three improvements were made in our research: (i) A deep learning-based stereo matching network was utilized to predict the depth image based on binocular RGB images, supporting the generation of more robust and accurate depth data. (ii) A heterogeneous image registration method was presented to achieve image alignment between thermal infrared and RGB images so that the thermal infrared image was also aligned with the depth image. (iii) Studies have shown that the recognition of object classes with a similar depth appearance and location is improved by combining depth information [51]. In our research, the depth image was added to the semantic segmentation network to improve the accuracy.

In summary, the objective of this article was to develop an approach for feather damage assessment based on visible light and infrared thermography. Fusing the information obtained from these two bands permitted a more reliable assessment of feather damage. The main contributions are as follows: (i) A novel pipeline was proposed to reconstruct the RGB-Depth-Thermal maps of chickens using binocular color cameras and a thermal infrared camera. (ii) A depth extraction algorithm for feather damage assessment was developed to determine the depth of feather damage from the RGB-Depth-Thermal model. (iii) The performance of the feather damage monitoring system was evaluated, for both day and night.

## 2. Materials and Methods

### 2.1. Experiment Materials and Image Acquisition

**Figure 1:**
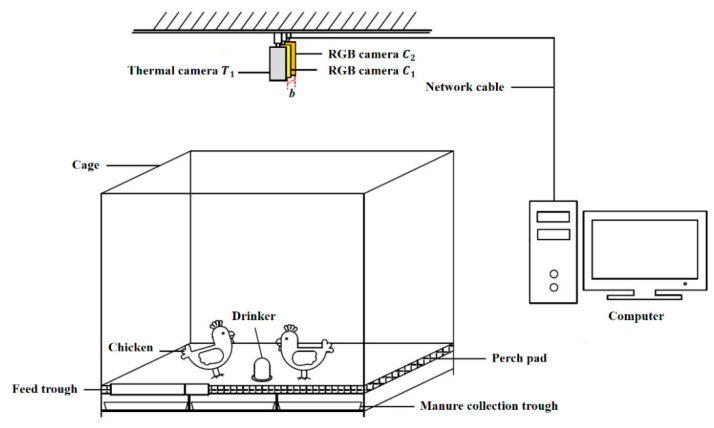
Experimental setup.

A binocular vision system was constructed in this experiment (Figure 1). Two dual-spectral thermography cameras (Hikvision, DS-2TD2636-10, 8~14 μm, uncooled core, China) were suspended 145 cm above the floor. The field of view was 87 cm × 47 cm on the ground. An RGB camera (6 mm, 1920 × 1080 pixels) and a thermal infrared camera (10 mm, 384 × 288 pixels) were included in a dual-spectral thermography camera. The baseline distance *b* between the binocular RGB cameras was 11.1 cm. The binocular RGB images and the left thermal infrared images were adopted for a 3D reconstruction of chicken images.

The image acquisition system was connected to a computer (AMD Ryzen 9 4900HS, 3.00 GHz per core, NVIDIA GeForce RTX 2060 GPU, 16GB RAM) for collecting images. The model was trained and inferred with a server equipped with a NVIDIA TITAN RTX GPU. “Python” was used as the programming language and “PyCharm” as the integrated development environment for image processing.

The experiment was conducted in Huzhou City, Zhejiang Province, PR China, on 8 January 2022 (moderate cold, 7–16 °C) and from 25 June 2022 to 3 July 2022 (moderate heat, 28–35 °C). Two batches of Chinese local yellow-feathered laying hen breed, including eight 24-week-old and twenty 20-week-old chickens, were collected separately. Usually, these hens were kept free-range on a thickly bedded floor. Only when collecting data were they placed in a separate cage, with dimensions of 145 cm × 97 cm × 130 cm (L × W × H). The cage was equipped with a drinker, a feed trough and a manure collection trough.

A total of 1000 sets of images (binocular RGB images and left thermal infrared images) of twenty 20-week-old chickens were chosen as a dataset for the evaluation of the disparity prediction and semantic segmentation performance. A total of 60%, 20% and 20% of the dataset were divided for training, validation and testing, respectively. To evaluate the performance of 3D reconstruction, the body length and body width of twenty 20-week-old chickens were measured manually and using our method, respectively. The ground truth of all the body sizes were measured using a measuring tape.

To simulate the feather damage of chickens, the feathers of 15 chickens (eight 24-week-old and seven 20-week-old chickens) were cut on the back area. The ground truth of damage depth was measured by a caliper. A total of 20 images for each chicken in different positions were selected for the evaluation of feather damage detection. The mean values of estimated damaged depth from 20 images were calculated as the predicted values of damaged depth for each chicken.

### 2.2. Methodology

**Figure 2:**
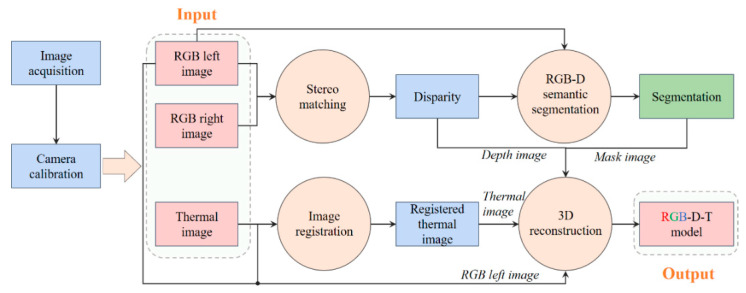
The pipeline of 3D reconstruction of chickens with RGB-D-T maps for feather damage monitoring. RGB-D-T: RGB-Depth-Thermal.

The overall pipeline of 3D reconstruction of chicken images for feather damage monitoring is shown in Figure 2. It consisted of the following steps:

1. Image acquisition (binocular RGB images and thermal infrared images);
2. Camera calibration of binocular RGB cameras and a thermal infrared camera;
3. Stereo matching for disparity prediction based on binocular RGB images;
4. RGB-D semantic segmentation based on the left RGB image and the predicted depth image;
5. Image registration of the left RGB image and the thermal infrared image;
6. Point clouds fusion between the color point clouds and the thermal infrared point clouds. The color point clouds were reconstructed by the left RGB image and the depth image, and the thermal infrared point clouds were reconstructed by the thermal infrared image and the depth image.

The following sections explain each step in more details.

#### 2.2.1. Camera Calibration

**Figure 3:**
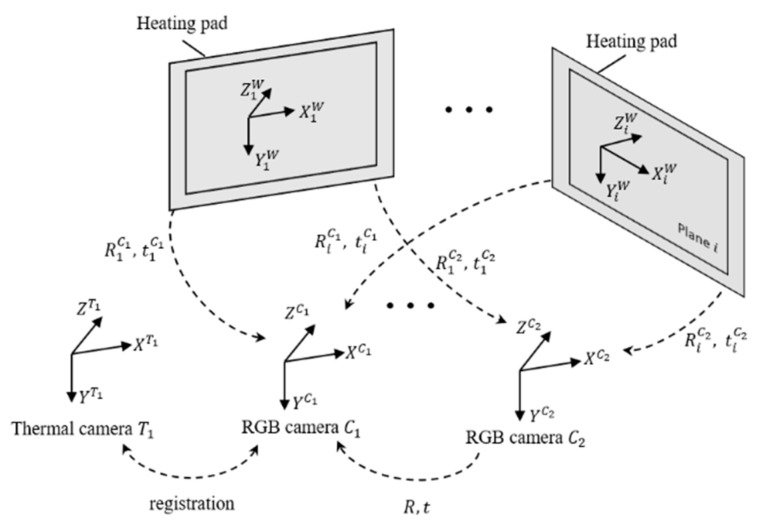
Camera calibration. $O^{C_{1}}-X^{C_{1}}Y^{C_{1}}Z^{C_{1}}$,$\;O^{C_{2}}-X^{C_{2}}Y^{C_{2}}Z^{C_{2}}$ and $O^{T_{1}}-X^{T_{1}}Y^{T_{1}}Z^{T_{1}}$ refer to the camera coordinate systems of RGB camera $C_{1}$, RGB camera $C_{2}$ and thermal camera $T_{1}$, respectively. “R” means the rotation matrix between two camera coordinate systems. “t” means the translation vector between two camera coordinate systems.

The position relationships between the binocular RGB cameras and a thermal infrared camera were determined through camera calibration (Figure 3). The Zhang Zhengyou calibration method [52] was utilized. A 15 × 16 black-white planar checkerboard (25 mm × 25 mm each cell) made of soda glass (white area) and chrome film (black area), was used as a calibration board. A heating plate (750 mm × 450 mm, set to 42 °C) was attached to the bottom of the calibration board. The white and black areas of the board were clearly distinguished due to the difference in the thermal properties of the two materials. The chrome film has a higher transmission and lower emissivity compared with the soda glass. Twenty-three groups of checkboard images from the three cameras were captured to calculate the internal and external parameters of the three cameras utilizing the MATLAB calibration toolbox [53]. The camera coordinate systems of RGB camera $C_{1}$, RGB camera $C_{2}$ and thermal camera $T_{1}$ are $O^{C_{1}}-X^{C_{1}}Y^{C_{1}}Z^{C_{1}}$, $O^{C_{2}}-X^{C_{2}}Y^{C_{2}}Z^{C_{2}}$ and $O^{T_{1}}-X^{T_{1}}Y^{T_{1}}Z^{T_{1}}$, respectively. The rotation matrix *R* and translation vector *t* between two camera coordinate systems were calculated as external parameters. The overall mean error of binocular cameras’ calibration was 0.33 pixels. The intrinsic parameters obtained from the camera calibration step were used as a reference for subsequent heterogenous image alignment and disparity prediction.

#### 2.2.2. Disparity Prediction

Adaptive aggregation network (AANet) is a state-of-art and efficient stereo matching network proposed in the 2020′s conference on computer vision and pattern recognition [54], which balances a fast inference speed and comparable accuracy. AANet was used for the disparity prediction of chicken images.

**Figure 4:**
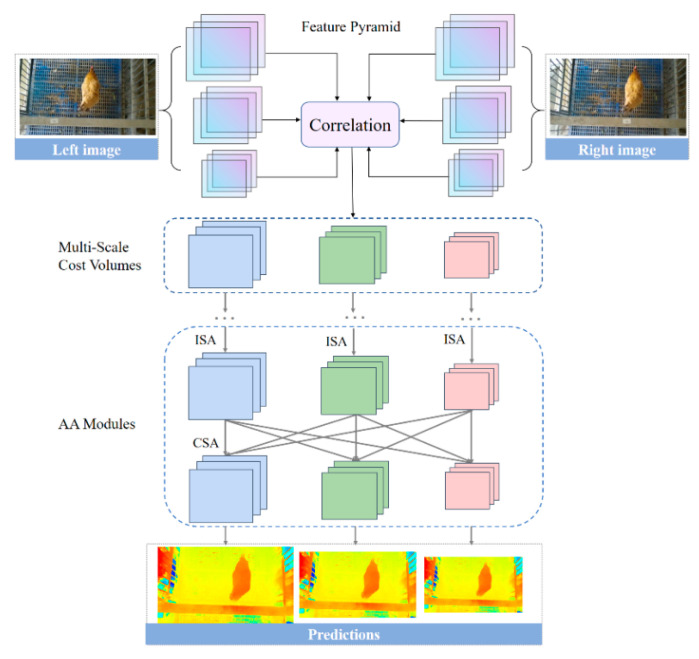
Overview structure of AANet for disparity prediction of chicken images. AANet: adaptive aggregation network. ISA: Intra-Scale Aggregation. CAS: Cross-Scale Aggregation. AA Module: Adaptive Aggregation Module. Each AA Module consisted of three ISA and a CSA.

Figure 4 provides the overview structure of AANet for disparity prediction of chicken images. Given a rectified RGB image pair of a chicken, we first extracted the down-sampled feature pyramid at 1/3, 1/6 and 1/12 resolutions by a shared feature extractor. The multi-scale 3D cost volumes [55] were constructed by correlating the features of the left and right images at corresponding scales (1/3, 1/6 and 1/12 resolutions). The cost volumes were aggregated with six stacked Adaptive Aggregation Modules (AA Modules). Each AA Module consisted of three Intra-Scale Aggregation (ISA) and a Cross-Scale Aggregation (CSA). The multi-scale disparity predictions were regressed by the soft argmin mechanism [56]. The final disparity prediction was hierarchically up-sampled and refined to the original resolution [57].

The dataset was augmented by random color augmentations and vertical flipping. The pre-trained AANet model for Scene Flow dataset [54] was used for direct inference on our dataset. The initial learning rate of the pre-trained AANet model was 0.001 and decreased by half at 400th, 600th, 800th and 900th epochs. Adam [58] was used to optimize the parameters of the network to minimize the average loss of the model on the training data. The disparity range was from 0 to 192 pixels.

#### 2.2.3. RGB-D Semantic Segmentation

**Figure 5:**
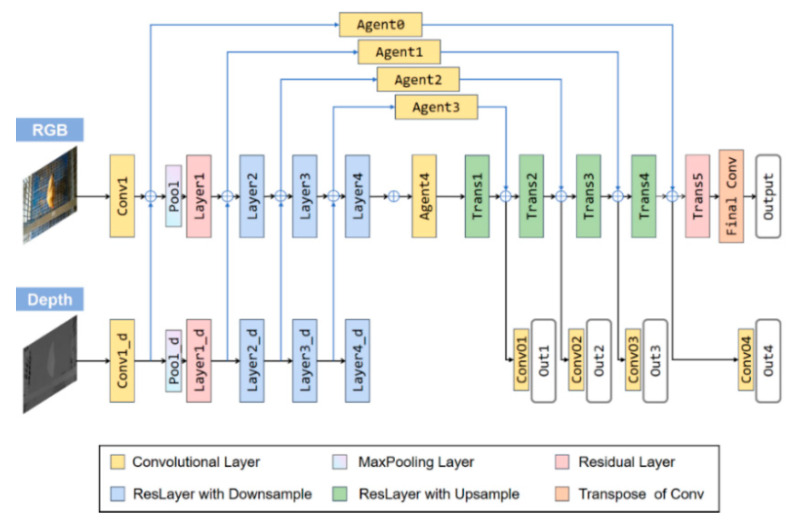
Overview structure of RedNet (ResNet-50) for semantic segmentation of chicken. RedNet (ResNet-50): residual encoder–decoder network using ResNet-50 as the basic feature extractor. ResNet-50: residual network with 50 layers.

Residual encoder–decoder network (RedNet) is a semantic segmentation network of excellent performance proposed by Jiang et al. [59]. It was utilized to improve the segmentation results by complementing the depth information to RGB signals. The RedNet structure is illustrated in Figure 5. The encoder–decoder network structure [48] was utilized in the RedNet network and the residual block was used as the building module. A pyramid supervision training scheme [59] was proposed to optimize the network. The pyramid supervision training scheme applied supervised learning over different layers in the decoder to avoid the gradients vanishing. Two convolutional branches, the RGB branch and the depth branch were included in the encoder structure, which had the same network configuration, except for the feature channel number of the convolution kernel. The element-wise summation was used for feature fusion.

During training, the dataset was augmented from 600 to 60,000 groups by applying random scale and crop, followed by random hue, brightness and saturation adjustment. When the epoch reached to approximately 100, the model had been converged. Stochastic gradient descent (SGD) was used to optimize the parameters of the network. The initial learning rate of SGD was set to 0.002. The target mask image could be segmented from the background by inferring.

#### 2.2.4. Thermal-RGB Image Registration

A method was proposed to achieve the thermal-RGB image registration within a fixed range distance based on checkerboard images. In general, the thermal infrared image was registered to RGB image through two steps: (i) scale and position adjusting and (ii) projection transformation. As the high-resolution RGB image would be time-consuming to reconstruct, it was resized from 1920 × 1080 to 960 × 540 pixels using the bilinear interpolation algorithm [60]. The focal length and principal point of the thermal infrared image were adjusted to be the same as that of the RGB image. In this way, the difference in image resolution and the principal point of the thermal infrared and the RGB images can be eliminated [61].

Then, the same corner points of the checkerboard pattern in the thermal infrared and RGB images were extracted for image pairing. The M-Estimate Sample Consensus (MSAC) algorithm [62] was utilized to eliminate incorrect matching pairs and select the optimal corresponding pairs. The projection matrix applied to convert the thermal infrared image coordinates ($\hat{x}$, $\hat{y}$) to the RGB image coordinates (*X*, *Y*) was as follows:

(1) $$\left[\begin{array}{c} X\\ Y\\ 1 \end{array}\right]=\left[\begin{array}{ccc} a_{1} & a_{2} & a_{3}\\ a_{4} & a_{5} & a_{6}\\ a_{7} & a_{8} & 1 \end{array}\right]\left[\begin{array}{c} \hat{x}\\ \hat{y}\\ 1 \end{array}\right]$$

where $a_{i}$(*i* = 1, 2, …, 8) are the parameter values to be calculated. Four corresponding pairs of (*x*, *y*) and (*X*, *Y*) must be known to solve Equation (1). Specifically, 4830 pairs of points extracted from checkerboard images were used to calculate the transformation matrix within a fixed-range distance, about 1.2~1.8 m.

**Figure 6:**
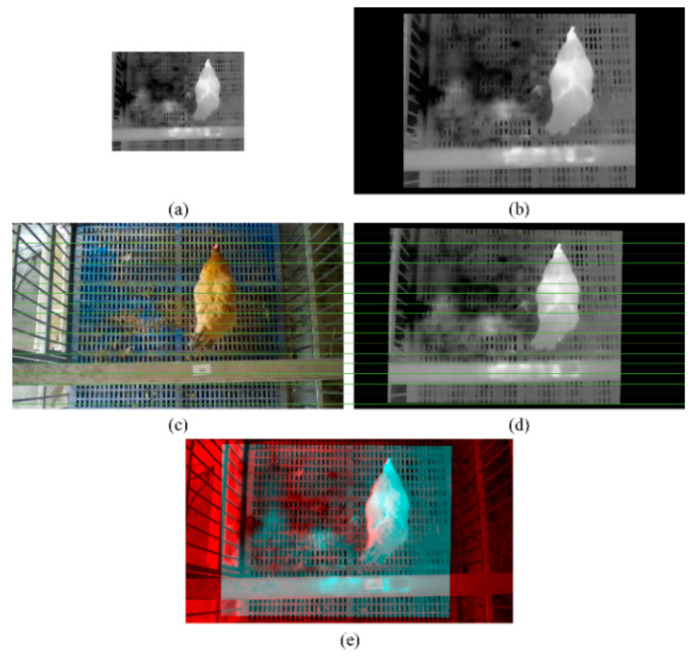
The process of the image registration between RGB and thermal infrared images: (**a**) original thermal infrared image; (**b**) thermal infrared image after scale and position adjusting of image (**a**); (**c**) original RGB image; (**d**) thermal infrared image after projection transformation of image (**b**); (**e**) red-cyan anaglyph of images (**c**) and (**d**).

The scale and position of the original thermal infrared image (Figure 6a) were adjusted to obtain the first transformation image (Figure 6b). Through projection transformation, the final thermal infrared image was achieved (Figure 6d). To better display the effect of image registration between the RGB and thermal infrared images, 18 green parallel lines were drawn on the RGB image (Figure 6c) and thermal infrared image (Figure 6d). The key points of the RGB image and thermal infrared image were row-aligned. The fused red-cyan anaglyph from the RGB image and the thermal infrared image are shown in Figure 6e. Each pixel of two images basically overlapped. Additionally, the reprojection errors of image registration were calculated based on 4830 pairs of corner points extracted from the checkerboard images. The results showed that the mean absolute error of the image registration was 1.25 pixels, while the root mean square error was 1.76 pixels, which demonstrates the effectiveness of the proposed image registration method.

#### 2.2.5. Three-Dimensional Reconstruction

**Figure 7:**
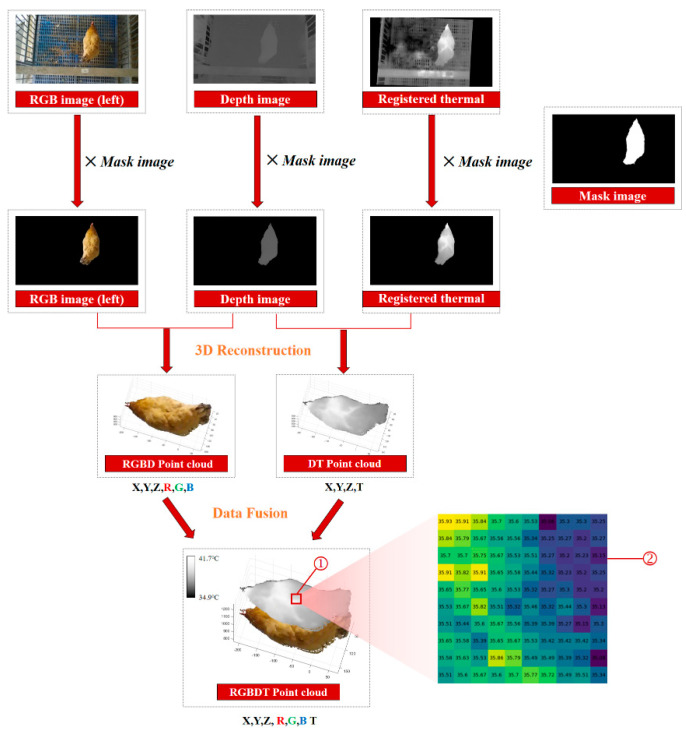
The example of 3D reconstruction with RGB-D-T maps for chicken. RGB-D-T: RGB-Depth-Thermal. The matrix ② showed the temperature value of each pixel of area ①.

The 3D point cloud of a chicken was reconstructed using the information obtained in the previous steps. An example of a 3D reconstruction with RGB-D-T maps of the chicken is shown in Figure 7. Four kinds of images, including the left RGB image, depth image, registered thermal infrared image and mask image, were utilized as inputs. The following steps were implemented for 3D reconstruction:

(i) The RGB and the mask images, the depth and the mask images, and the registered thermal infrared and the mask images, were processed using bitwise and operations [63], respectively;
(ii) The RGB image was combined with the depth image to obtain the target color point clouds, and the registered thermal infrared image was combined with the depth image to obtain the target temperature point clouds;
(iii) The color point clouds and the temperature point clouds were fused to obtain the final RGB-D-T model of chicken by dimensional expansion.

A multi-dimensional vector was created to store this model. One point of the RGB-D-T point cloud can be described as: *P* {*x*, *y*, *z*, *r*, *g*, *b*, *t*}, where {*x*, *y*, *z*} represent the spatial coordinates at the point *P*, {*r*, *g*, *b*} represent the color values of the three channels at the point *P*, and *t* represents the temperature at the point *P*. For a better display, the z-coordinate value of each point of the thermal point cloud was increased by 300 mm and merged these two point clouds. The matrix ② in Figure 7 showed the temperature value of each pixel of area ①.

### 2.3. Feather Damage Detection and Depth Estimation

To better display the feather damage region, all the thermal infrared images were acquired in a pseudo-color mode and processed based on the mask images in Section 2.2.5. The feather of a chicken was judged whether it was damaged or not. If the feather was damaged, the feather damage region was extracted and the damage depth was estimated. The total pipeline can be divided into the following two steps:

Step 1: Feather damage detection and extraction

**Figure 8:**
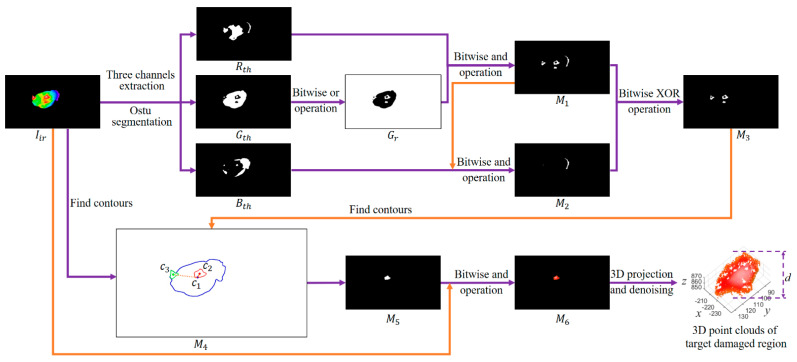
The flow diagram of feather damage detection and extraction. $I_{ir}$: the thermal infrared image of a chicken. $R_{th}$, $G_{th}$ and $B_{th}$: the three channels of the thermal infrared image $I_{ir}$ processed with the Ostu segmentation algorithm. $G_{r}$: the image processed with bitwise or operation from the image $G_{th}$. $M_{1}$: the image processed with bitwise and operation from the images $G_{r}$ and $R_{th}$. $M_{2}$: the image processed with bitwise and operation from the images $M_{1}$ and $B_{th}$. $M_{3}$: the image processed with bitwise exclusion-or (XOR) operation from the images $M_{1}$ and $M_{2}$. $M_{4}$: the schematic diagram of the contours and centroid points extraction from the images $I_{ir}$ and $M_{3}$. The centroid points of the top two largest contours in image $M_{3}$ were extracted, which labeled as $c_{2}$ and $c_{3}$, respectively. The centroid point of the contour in image $I_{ir}$ was also extracted and labeled as $c_{1}$. $M_{5}$: the target binary image with feather-damaged zone. $M_{6}$: the image processed with bitwise and operation from the images $I_{ir}$ and $M_{5}$. The image $M_{6}$ was projected to a 3D coordinate system. A clean point cloud of the damaged region could be acquired after denoising. The difference value *d* between the minimum and the maximum z coordinate values of the target point clouds was calculated as the predicted depth of the feather-damaged region.

The algorithm for the feather damage detection and extraction is described in Figure 8. Firstly, the three channels of the thermal infrared image $I_{ir}$ in RGB color space were extracted and processed with the Ostu segmentation algorithm [64] to obtain segmented binary images $R_{th}$, $G_{th}$ and $B_{th}$, respectively. Secondly, the image $G_{th}$ was processed with bitwise or operation [63] to obtain image $G_{r}$. The images $G_{r}$ and $R_{th}$ were processed with bitwise and operation to obtain image $M_{1}$. The images $M_{1}$ and $B_{th}$ were processed with bitwise and operation to obtain image $M_{2}$. The images $M_{1}$ and $M_{2}$ were processed with bitwise exclusion-or (XOR) operation [65], which returns a true value when either but not both of its operands is true, to obtain image $M_{3}$. Thirdly, the number of the contours in potential target regions was used as the criterion to judge whether the feather of the chicken was damaged or not. Since the head region of chicken was also featherless, the temperature of the head region was very close to the damaged region, which disturbed the detection of the damaged region. According to the number of the contours in image $M_{3}$, the results of feather damage detection can be divided into the following three situations: (i) If the number of contours in image $M_{3}$ was 0, the feather of the chicken was judged as not damaged. (ii) If the number of contours in image $M_{3}$ was 1 and the pixel area of the contour was more than 300, the feather of the chicken was judged as damaged and the region of the contour in binary image $M_{5}$ was the target feather-damaged zone. Otherwise, the feather of the chicken was judged as not damaged. (iii) If the number of contours in image $M_{3}$ was 2 or more, the centroid points of the top two largest contours in image $M_{3}$ were extracted, which labeled as $c_{2}$ and $c_{3}$, respectively. The centroid point of the contour in image $I_{ir}$ was also extracted and labeled as $c_{1}$. By comparing the Euclidean distance between $c_{1}$ and $c_{2}$, and the Euclidean distance between $c_{1}$ and $c_{3}$, the potential target region in image $M_{4}$ can be obtained by choosing the contour with the shorter distance. If the pixel area of potential target region in image $M_{4}$ was more than 300, the feather of the chicken was judged as damaged and the region of the contour in binary image $M_{5}$ was the target damaged zone. Otherwise, the feather of the chicken was judged as not damaged.

Step 2: Feather damage depth estimation

After obtaining the target binary image $M_{5}$, the images $I_{ir}$ and $M_{5}$ were processed with bitwise and operation to obtain image $M_{6}$, which only contained the feather-damaged region with color information. Since the 3D point cloud was reconstructed from 2D images, every 2D mask on the 2D images can be projected to a unique 3D patch on the 3D point clouds. Therefore, the image $M_{6}$ was projected to a 3D coordinate system. To eliminate the influence of segmented edge points on depth extraction, the sparse points were removed by the Radius-Outlier-Removal filter [66]. A clean point cloud of the damaged region could be acquired after denoising. The difference value *d* between the minimum and the maximum z coordinate values of the target point clouds was calculated as the predicted depth of the feather-damaged region.

### 2.4. Performance Metrics

To evaluate the effect of feather damage depth estimation, the coefficient of determination ($R^{2}$) and the root mean square error (*RMSE*) of feather damage depth were calculated based on the proposed model and manual measurement method.

Additionally, the pixel accuracy (*PA*), intersection-over-union (*IoU*), model size and inference speed were used to evaluate the performance of the segmentation network. The *PA* was the ratio of the number of pixels correctly identified to the total number of pixels in the test set. The *IoU* was the ratio of the intersection and the concatenation of the two sets, which refers to the ground truth and the predicted segmentation. The *PA* and *IoU* were calculated as:

(2) $$PA=\frac{\sum_{i=0}^{k}p_{ii}}{\sum_{i=0}^{k}\sum_{j=0}^{k}p_{ij}}$$

(3) $$IoU=\frac{1}{k+1}\sum_{i=0}^{k}\frac{p_{ii}}{\sum_{j=0}^{k}p_{ij}+\sum_{j=0}^{k}p_{ji}-p_{ii}}$$

where *k* is the number of categories containing empty classes (here *k* = 1), *p_ii_* is the number of true values of *i* and predicted to be *i*, *p_ij_* is the number of true values of *i* that are predicted to be *j*, and *p_ji_* is the number of true values of *j* that are predicted to be *i*.

## 3. Results and Discussion

### 3.1. 3D Reconstruction Results of Chicken

**Figure 9:**
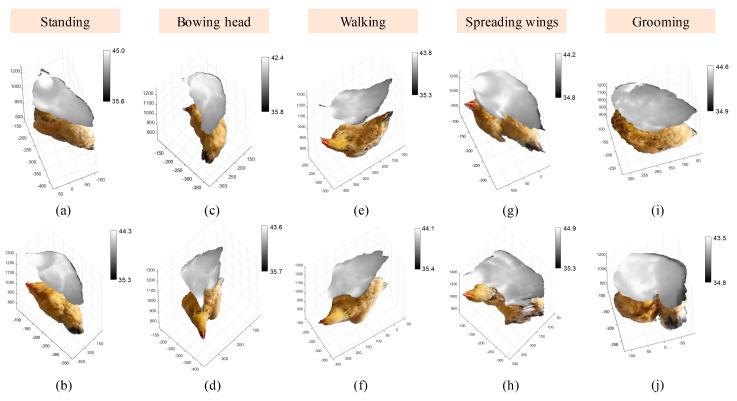
RGB-D-T point clouds for five postures of chicken generated by the proposed 3D reconstruction method: (**a**,**b**) “standing” posture; (**c**,**d**) “bowing head” posture; (**e**,**f**) “walking” posture; (**g**,**h**) “spreading wings” posture; (**i**,**j**) “grooming” posture. RGB-D-T: RGB-Depth-Thermal.

**Figure 10:**
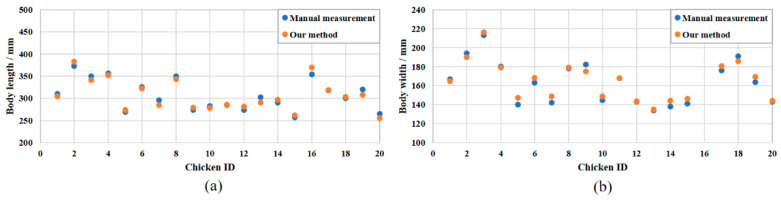
Comparison of the manual measurement method and our method for the: (**a**) body length; and (**b**) body width.

To evaluate the reconstruction effect of chickens, the 3D reconstruction results for chickens were divided into five postures: “standing”, ”bowing head”, ”walking”, ”spreading wings” and ”grooming” (Figure 9). The reconstructed model displayed the color and thermal information of chickens in different postures with great accuracy and low noise. The edges of the chicken were well recognized and segmented from the background. Figure 10 shows the comparison of the manual measurement method and our method for the body length and body width. The manual body size measurement was used as the reference to evaluate the measurement results in our method. The selected chicken images using in our method were in “standing” posture. From the data in Figure 10, it can be calculated that the mean relative errors of body length and body width between the manual measurement method and our method for 20 chickens were 2.20% and 2.39%, respectively. The results of Figure 9 and Figure 10 demonstrated that the RedNet-based semantic segmentation method and the AANet-based stereo matching method were effective for the reconstruction of feathers.

Table 1 lists the mean values of temperatures for the head, back and tail areas of chickens under different ambient temperatures. Two different ambient environments, including moderate heat (MoH, 28–35 °C) and moderate cold (MoC, 7–16 °C), were considered. The highest surface temperature of the chickens was the head area, which was featherless, and the lowest surface temperature of the chickens was the back or tail area, which was fully feathered. The results were consistent with the previous studies [67].

When there was a significant increase in the body surface temperature (feather damage or inflammation process), this could be identified by our developed method and measures can be taken to avoid economic losses. If the system detected a body abnormality, the developed system could serve as a monitoring tool.

### 3.2. Evaluation of Feather Damage Depth Estimation

**Figure 11:**
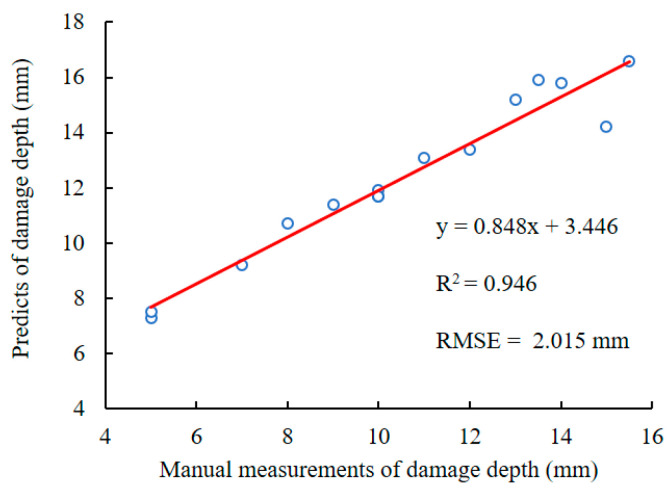
Linear regression for feather damage depth based on the predicted results with manual measurements.

Linear regression for feather damage depth was conducted on predicted values regressed with manual measurements of 15 feather-damaged chickens (Figure 11). The results showed that the $R^{2}$ was 0.946 with an *RMSE* of 2.015 mm. Overall, the depth of feather damage from the proposed method was highly correlated with manual measurement method. However, there were still some reasons for errors in depth estimation. First, the error of disparity prediction based on binocular RGB images may have a significant effect on depth estimation of feather damage. If the color and texture of the damaged region and the non-damaged region in the RGB image were similar, the predicted depth of feather damage would be underestimated. Second, the posture of the target chicken was another uncertain factor for damage depth estimation. For example, if the chicken was in the posture of “bowing head”, “spreading wings” or “grooming”, the estimated depth of feather damage would be affected. Future work could focus on the ideal posture recognition of chicken for precise feather damage monitoring.

### 3.3. Feather Damage Monitoring Based on the Proposed Method vs. 2D Thermal Infrared Image or RGB Image

**Figure 12:**
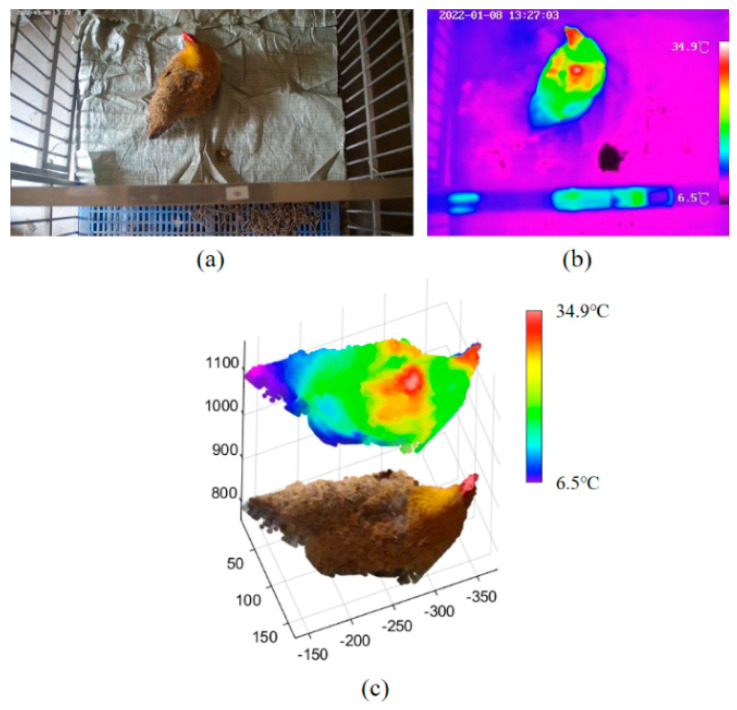
Sample of chicken point clouds with feather damage: (**a**) RGB image with feather damage; (**b**) thermal infrared image with feather damage; (**c**) RGB-D-T point clouds with feather damage. RGB-D-T: RGB-Depth-Thermal.

The point clouds of sample chicken with feather damage on the back were reconstructed (Figure 12). The thermal infrared image was acquired in a pseudo-color mode. The experimental data showed that, compared with 2D RGB image (Figure 12a) or thermal infrared image (Figure 12b), the depth of the damage region can be measured from the proposed RGB-D-T model (Figure 12c), which was more intuitive and comprehensive for feather damage assessment. The feather damage was difficult to observe from an RGB image (Figure 12a) when the depth of the damage region was not deep. Additionally, the depth of feather damage was uncertain from the thermal infrared image (Figure 12b), even though the feather damage could be identified.

**Figure 13:**
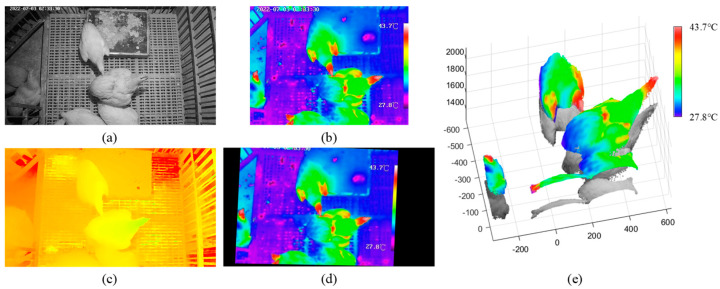
Example of chicken point clouds with feather damage at night: (**a**) RGB image with feather damage at night; (**b**) thermal infrared image with feather damage at night; (**c**) depth image predicted by binocular RGB images at night; (**d**) thermal infrared image with feather damage at night after image registration; (**e**) RGB-D-T point clouds with feather damage at night. RGB-D-T: RGB-Depth-Thermal.

In addition to the detection effect of feather damage in the daytime, the situation of darkness at night was also validated (Figure 13). It can be found that the detection of feather damage was affected by the weak texture and low contrast of the RGB image (Figure 13a), which made it difficult to distinguish the region and size of feather damage. However, the depth image at night can also be predicted by our model (Figure 13c). Additionally, through image registration, the thermal infrared image (Figure 13d) can be fused with the RGB image to obtain the RGB-D-T model. A clean 3D model of chickens was obtained (Figure 13e) after utilizing distance filtering. There was a significant high-temperature region on the back of the reconstructed RGB-D-T model, and the depth of the feather damage can be obtained.

The results demonstrated that the RGB-D-T 3D model was a promising tool for the feather damage assessment of chickens. In contrast to previous works [19,23], the approach was no longer limited to 2D thermal infrared images, but mapped the thermal images to 3D point clouds to extract the depth of the damage region. The combined video data from the binocular RGB camera and thermal imaging camera provided a method for an almost complete monitoring of chickens during both day and night.

### 3.4. Evaluation of Chicken Disparity Prediction

**Figure 14:**
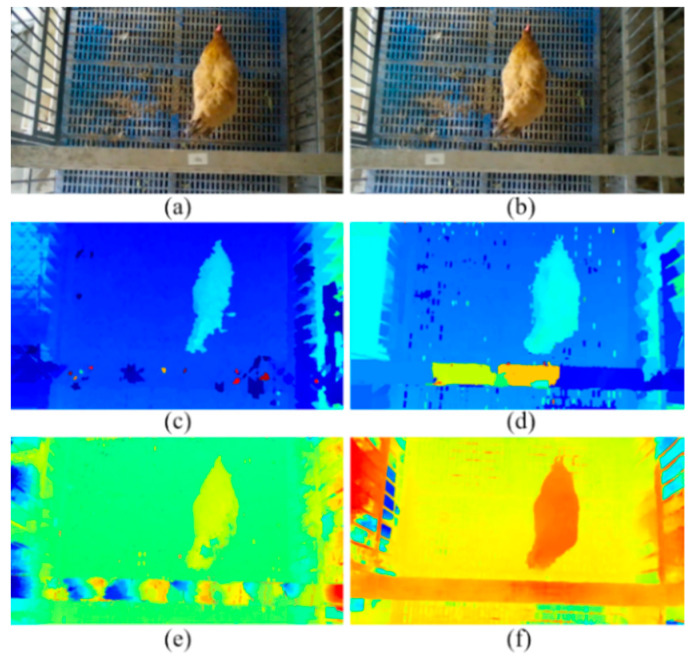
Effect comparison of different stereo matching algorithms: (**a**) left rectified RGB image; (**b**) right rectified RGB image; (**c**) SGBM; (**d**) AD-Census; (**e**) PMS; (**f**) AANet. SGBM: semi-global block matching. AD-Census: absolute differences measure and census transform. PMS: PatchMatch stereo. AANet: adaptive aggregation network.

The AANet algorithm was compared with three classical stereo algorithms: semi-global block matching (SGBM) [68], absolute differences measure and census transform (AD-Census) [69] and PatchMatch stereo (PMS) [70]. The disparity map of different algorithms (Figure 14) was obtained through the left rectified RGB image (Figure 14a) and the right rectified RGB image (Figure 14b). All the disparity maps were transformed into pseudo-color images to compare the disparity prediction accuracy. The disparity map obtained by the AANet model was the best compared to the other three algorithms, with more accurate contour details and better predictions of the objects, especially towards the railing. Referring to the inference speed, the four algorithms were all tested on the remote server with a NVIDIA TITAN RTX GPU. The inference speeds of the SGBM, AD-Census, PMS and AANet model were 4.000 fps, 0.157 fps, 0.007 fps and 2.037 fps, respectively. The fastest inference speed was the SGBM algorithm, but this had the worst disparity map effect. The PMS algorithm required the longest inference time, which was mainly due to the huge computation required to estimate the individual 3D plane at each pixel. It can be easily found that the deep learning-based AANet model achieved a better balance between accuracy and inference speed compared with SGBM, AD-Census and PMS methods. It was fully feasible to utilize the AANet algorithm for the stereo matching and disparity prediction of chicken images.

### 3.5. Evaluation of Chicken Semantic Segmentation Based on Color and Depth Images

**Figure 15:**
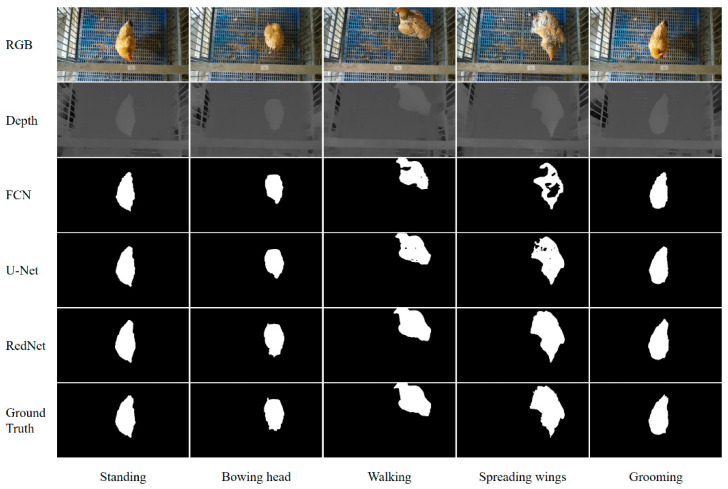
Qualitative comparison of the RedNet (ResNet-50) and the recent competitive semantic segmentation methods. RedNet (ResNet-50): residual encoder–decoder network using ResNet-50 as the basic feature extractor. ResNet-50: residual network with 50 layers. FCN: fully convolutional network. U-Net: a network that the architecture looks like the letter “U”.

The FCN and U-Net were used as comparison networks to verify the effectiveness of the RedNet used in our pipeline. The segmented ground truth for each image at the pixel level was obtained using the LabelMe toolbox [71]. A comparison of segmentation results for FCN, U-Net and RedNet is shown in Figure 15.

To evaluate the robustness of segmentation for different postures, the testing set was classified into five poses, including “standing”, “bowing head”, “walking”, “spreading wings” and “grooming”. The segmentation effect of RedNet was the best compared to FCN and U-Net, especially in the darker region of the feather and contour details (Figure 15). The results suggested that the RedNet algorithm was more robust to different poses compared to FCN and U-Net.

The comprehensive performance metrics for the dataset of 1000 images on three semantic segmentation algorithms are listed in Table 2. In addition to *IoU*, the metric of *PA*, model size and inference time were also calculated. The *PA* and *IoU* of RedNet both had the highest values compared to FCN and U-Net, with 0.997 and 0.978, respectively. It was confirmed that the segmentation accuracy was significantly improved by complementing disparity images. In addition, three algorithms were all tested on the remote server with a NVIDIA TITAN RTX GPU to compare the inference speed. It was found that RedNet had the slowest inference speed (17.857 fps) and the largest model size (313 MB) among the three models. This was mainly due to the combination of RGB and depth branches used for training. Nevertheless, the inference speed of RedNet exceeded 15 fps, which indicated that utilizing RedNet could already meet the real-time requirements.

### 3.6. Time Efficiency Analysis

For daily monitoring of poultry, time efficiency was necessary to be taken into account. The time efficiency was analyzed on the test set and shown in Table 3. The longest subtask time was disparity prediction based on binocular RGB images, which was mainly due to the complexity of the AANet model. The average run-time and the standard deviation of a set of images were 0.627 s and 0.149 s, respectively. This demonstrates the feasibility of real-time monitoring and the stability of the method.

### 3.7. Limitations and Future Works

**Figure 16:**
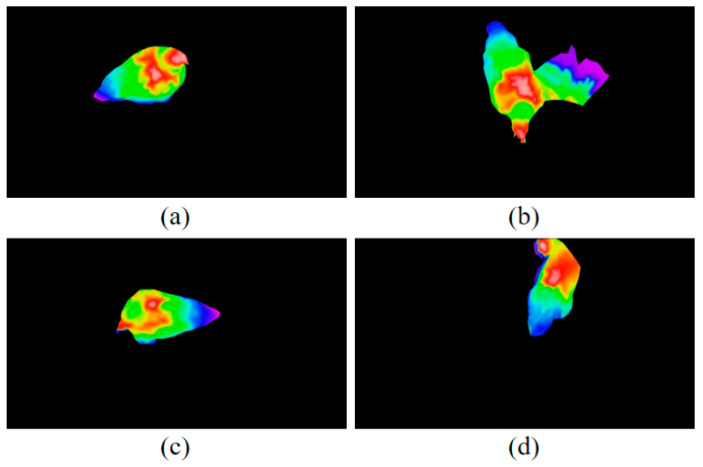
Thermal infrared images of chicken in different postures. (**a**) “standing” posture; (**b**) “spreading wings” posture; (**c**) “bowing head” posture; (**d**) “grooming” posture.

The results of our experiments demonstrated that the RGB-D-T model provides a potential tool for feather damage monitoring and assessment. Limitations can be found in our method. Since the experimental scenario in this paper was a simulated flat-rearing condition, only single chicken was shown in each image. If the proposed approach was applied in an actual commercial environment, multiple chickens will be involved and chickens may be occluded from each other, or by feeding or drinking equipment [72]. This would result in some chickens having incomplete point clouds when reconstructed, so that their feather cover condition could not be effectively assessed. In addition, the chickens’ postures may affect the feather damage recognition. For various poses, the size and the depth of the feather damage area identified from the images were different (Figure 16). In this paper, the chicken dataset was selected with ideal postures (“standing” posture).

Therefore, future works should focus on the robustness of the proposed model for the occlusion of multiple chickens and posture variation. An algorithm for multi-target video-tracking [73] could be finetuned to detect the posture of each moving chicken in a frame-by-frame tracking mode. If the posture of a chicken is judged as “standing” posture, the subsequent feather damage assessment can be conducted. This research will be implemented in a further study. Our system needs to solve the above problems to suit the practical environment and commercial applications.

## 4. Conclusions

In this study, a novel approach to feather damage monitoring was proposed based on visible light and infrared thermography. The results demonstrated that the proposed RGB-D-T model could detect the region of feather damage and assess the depth of feather damage. The main conclusions were as follows:

(1) A feather damage monitoring system was proposed using binocular RGB cameras and a thermal infrared camera. The depth image of the chicken was predicted using the AANet network based on binocular RGB images. The chicken image was segmented from the background utilizing the RedNet network based on the RGB image and depth image. The RGB image and thermal infrared image were registered by the proposed heterogenous image registration method. Four kinds of images, namely RGB, depth, thermal and mask, were utilized as inputs to reconstruct the 3D model of chicken with RGB-Depth-Thermal maps. The results showed that the deep learning-based AANet network was more efficient than the other three traditional stereo matching algorithms.
(2) Based on the obtained RGB-D-T model, an automated assessment algorithm for the depth of feather damage was developed. The feather damage region was extracted by image pre-processing based on the thermal infrared image. The feather damage region on the 2D images was projected to a unique 3D patch on the 3D point clouds. The depth value was calculated by the difference value between the minimum z-value and the maximum z-value of the target point clouds after filtering. The results showed that the $R^{2}$ was 0.946, with an *RMSE* of 2.015 mm between the predicted depth of feather damage and manual measurement.
(3) The feather damage monitoring system for chickens was tested during both day and night. This indicated that the proposed RGB-D-T model was more effective for feather damage detection than the 2D RGB image or thermal infrared image. The results provide ideas for future research on automation and intelligent feather damage monitoring in poultry farming.

## Figures and Tables

**Table 1:** Mean values of temperatures for the head, back and tail areas of chickens under different ambient temperatures.

Environment	Head Area (°C)	Back Area (°C)	Tail Area (°C)
MoH *	43.6 ± 0.9	39.2 ± 1.2	38.8 ± 1.0
MoC *	39.5 ± 1.9	35.4 ± 1.5	34.6 ± 1.3

* MoH refers to moderate heat (28–35 °C), and MoC refers to moderate cold (7–16 °C).

**Table 2:** Quantitative comparison of *PA*, *IoU*, model size and inference speed on three semantic segmentation methods.

Methods	*PA*	*IoU*	Model Size (MB)	Inference Speed (fps)
FCN	0.840	0.839	77	21.277
U-Net	0.921	0.919	30	21.739
RedNet	0.997	0.978	313	17.857

**Table 3:** Time cost statistics for each step of the overall pipeline.

Subtasks	Average Time (s)	Standard Deviation (s)
Disparity prediction	0.491	0.122
Semantic segmentation	0.056	0.008
Image registration	0.020	0.005
3D reconstruction	0.060	0.014
Total	0.627	0.149

## Data Availability

The data are not publicly available due to privacy reasons.

## Abbreviations

FS	Feather scoring
IRT	Infrared thermography
TOF	Time-of-flight
LiDAR	Structured light cameras or light detection and ranging
RGB-D-T	RGB-Depth-Thermal
ICP	Iterative Closest Point
FCN	Fully convolutional networks
AANet	Adaptive aggregation network
AA Modules	Adaptive Aggregation Modules
ISA	Intra-Scale Aggregation
CSA	Cross-Scale Aggregation
RedNet	Residual encoder–decoder network
ResNet-50	Residual network with 50 layers
SGD	Stochastic gradient descent
MSAC	M-Estimate Sample Consensus
XOR	Exclusion-or
$R^{2}$	Coefficient of determination
*RMSE*	Root mean square error
*PA*	Pixel accuracy
*IoU*	Intersection-over-union
MoH	Moderate heat
MoC	Moderate cold
SGBM	Semi-Global Block Matching
AD-Census	Absolute differences measure and census transform
PMS	PatchMatch Stereo

## References

[1] Buller H., Blokhuis H., Jensen P., Keeling L. (2018). Towards farm animal welfare and sustainability. Animals.

[2] Xu D., Shu G., Liu Y., Qin P., Zheng Y., Tian Y., Zhao X., Du X. (2022). Farm environmental enrichments improve the welfare of layer chicks and pullets: A comprehensive review. Animals.

[3] Tauson R., Kjaer J., Maria G., Cepero R., Holm K. (2005). Applied scoring of integument and health in laying hens. Anim. Sci. Pap. Rep..

[4] Savory C.J. (1995). Feather pecking and cannibalism. World’s Poult. Sci. J..

[5] Glatz P.C. (2001). Effect of poor feather cover on feed intake and production of aged laying hens. Asian-Australas. J. Anim. Sci..

[6] Bilcik B., Keeling L.J. (1999). Changes in feather condition in relation to feather pecking and aggressive behaviour in laying hens. Br. Poult. Sci..

[7] Spindler B., Weseloh T., Esser C., Freytag S.K., Klambeck L., Kemper N., Andersson R. (2020). The effects of UV-A light provided in addition to standard lighting on plumage condition in laying hens. Animals.

[8] Kjaer J.B., Sorensen P. (2002). Feather pecking and cannibalism in free-range laying hens as affected by genotype, dietary level of methionine + cystine, light intensity during rearing and age at first access to the range area. Appl. Anim. Behav. Sci..

[9] Savory C.J., Mann J.S., Macleod M.G. (1999). Incidence of pecking damage in growing bantams in relation to food form, group size, stocking density, dietary tryptophan concentration and dietary protein source. Br. Poult. Sci..

[10] Savory C.J., Mann J.S. (1999). Feather pecking in groups of growing bantams in relation to floor litter substrate and plumage colour. Br. Poult. Sci..

[11] El-Lethey H., Aerni V., Jungi T.W., Wechsler B. (2000). Stress and feather pecking in laying hens in relation to housing conditions. Br. Poult. Sci..

[12] Van de Weerd H.A., Elson A. (2006). Rearing factors that influence the propensity for injurious feather pecking in laying hens. World’s Poult. Sci. J..

[13] Newberry R.C., Keeling L.J., Estevez I., Bilcik B. (2007). Behaviour when young as a predictor of severe feather pecking in adult laying hens: The redirected foraging hypothesis revisited. Appl. Anim. Behav. Sci..

[14] Nichelmann M., Baranyiova E., Goll R., Tzschentke B. (1986). Influence of feather cover on heat balance in laying hens (Gallus domesticus). J. Therm. Biol..

[15] Decina C., Berke O., van Staaveren N., Baes C.F., Harlander-Matauscheck A. (2019). Development of a scoring system to assess feather damage in canadian laying hen flocks. Animals.

[16] Van Staaveren N., Ellis J., Baes C.F., Harlander-Matauschek A. (2021). A meta-analysis on the effect of environmental enrichment on feather pecking and feather damage in laying hens. Poult. Sci..

[17] Cronin G.M., Glatz P.C. (2021). Causes of feather pecking and subsequent welfare issues for the laying hen: A review. Anim. Prod. Sci..

[18] Tauson R., Ambrosen T., Elwinger K. (1984). Evaluation of procedures for scoring the integument of laying hens—Independent scoring of plumage condition. Acta Agric. Scand..

[19] Cook N.J., Smykot A.B., Holm D.E., Fasenko G., Church J.S. (2006). Assessing feather cover of laying hens by infrared thermography. J. Appl. Poult. Res..

[20] Zhao Y., Xin H., Dong B. (2013). Use of infrared thermography to assess laying-hen feather coverage. Poult. Sci..

[21] Mota-Rojas D., Titto C.G., de Mira Geraldo A., Martinez-Burnes J., Gomez J., Hernandez-avalos I., Casas A., Dominguez A., Jose N., Bertoni A. (2021). Efficacy and function of feathers, hair, and glabrous skin in the thermoregulation strategies of domestic animals. Animals.

[22] Cilulko J., Janiszewski P., Bogdaszewski M., Szczygielska E. (2013). Infrared thermal imaging in studies of wild animals. Eur. J. Wildl. Res..

[23] Redaelli V., Ludwig N., Costa L.N., Crosta L., Riva J., Luzi F. (2014). Potential application of thermography (irt) in animal production and for animal welfare. A case report of working dogs. Ann. Ist. Super. Sanita.

[24] Casas-Alvarado A., Mota-Rojas D., Hernandez-Avalos I., Mora-Medina P., Olmos-Hernandez A., Verduzco-Mendoza A., Reyes-Sotelo B., Martinez-Burnes J. (2020). Advances in infrared thermography: Surgical aspects, vascular changes, and pain monitoring in veterinary medicine. J. Therm. Biol..

[25] McCafferty D.J. (2013). Applications of thermal imaging in avian science. Ibis.

[26] Barreto C.D., Alves F.V., Ramos C., Leite M.C.D., Leite L.C., Karvatte N. (2020). Infrared thermography for evaluation of the environmental thermal comfort for livestock. Int. J. Biometeorol..

[27] Giersberg M.F., Spindler B., Kemper N. (2017). Assessment of plumage and integument condition in dual-purpose breeds and conventional layers. Animals.

[28] Nääs I.d.A., Bites Romanini C.E., Neves D.P., do Nascimento G.R., Vercellino R.d.A. (2010). Broiler surface temperature distribution of 42 day old chickens. Sci. Agric..

[29] Pichová K., Bilcik B., Kost’al L. (2017). Assessment of the effect of housing on feather damage in laying hens using IR thermography. Animal.

[30] Del Valle J.E., Pereira D.F., Mollo Neto M., Almeida Gabriel Filho L.R., Salgado D.D. (2021). Unrest index for estimating thermal comfort of poultry birds (Gallus gallus domesticus) using computer vision techniques. Biosyst. Eng..

[31] Okinda C., Lu M., Liu L., Nyalala I., Muneri C., Wang J., Zhang H., Shen M. (2019). A machine vision system for early detection and prediction of sick birds: A broiler chicken model. Biosyst. Eng..

[32] Pereira D.F., Miyamoto B.C.B., Maia G.D.N., Sales G.T., Magalhaes M.M., Gates R.S. (2013). Machine vision to identify broiler breeder behavior. Comput. Electron. Agric..

[33] Xiao L., Ding K., Gao Y., Rao X. (2019). Behavior-induced health condition monitoring of caged chickens using binocular vision. Comput. Electron. Agric..

[34] Paturkar A., Sen Gupta G., Bailey D. (2021). Making use of 3D models for plant physiognomic analysis: A review. Remote Sens..

[35] Susperregi L., Maria Martinez-Otzeta J., Ansuategui A., Ibarguren A., Sierra B. (2013). Rgb-d, laser and thermal sensor fusion for people following in a mobile robot. Int. J. Adv. Robot. Syst..

[36] Krishnan A.K., Saripalli S. (2017). Cross-calibration of rgb and thermal cameras with a lidar for rgb-depth-thermal mapping. Unmanned Syst..

[37] Chang M.-C., Yu T., Luo J., Duan K., Tu P., Zhao Y., Nagraj N., Rajiv V., Priebe M., Wood E.A. (2018). Multimodal sensor system for pressure ulcer wound assessment and care. IEEE Trans. Ind. Inform..

[38] Shi Y., Payeur P., Frize M., Bariciak E. Thermal and rgb-d imaging for necrotizing enterocolitis detection. Proceedings of the 2020 IEEE International Symposium on Medical Measurements and Applications.

[39] Lagüela S., Armesto J., Arias P., Herraez J. (2012). Automation of thermographic 3D modelling through image fusion and image matching techniques. Autom. Constr..

[40] Ham Y., Golparvar-Fard M. (2013). An automated vision-based method for rapid 3D energy performance modeling of existing buildings using thermal and digital imagery. Adv. Eng. Inform..

[41] Seitz S.M., Curless B., Diebel J., Scharstein D., Szeliski R. A comparison and evaluation of multi-view stereo reconstruction algorithms. Proceedings of the IEEE/CVF Conference on Computer Vision and Pattern Recognition.

[42] Hoegner L., Tuttas S., Xu Y., Eder K., Stilla U. (2016). Evaluation of methods for coregistration and fusion of RPAS-based 3D point clouds and thermal infrared images. ISPRS—Int. Arch. Photogramm. Remote Sens. Spat. Inf. Sci..

[43] Besl P.J., Mckay N.D. (1992). A method for registration of 3-D shapes. IEEE Trans. Pattern Anal. Mach. Intell..

[44] Di Stefano L., Marchionni M., Mattoccia S. (2004). A fast area-based stereo matching algorithm. Image Vis. Comput..

[45] Wu F., Fang X. An improved RANSAC homography algorithm for feature based image mosaic. Proceedings of the 7th WSEAS International Conference on Signal Processing, Computational Geometry & Artificial Vision.

[46] Lowe D.G. (2004). Distinctive image features from scale-invariant keypoints. Int. J. Comput. Vis..

[47] Long J., Shelhamer E., Darrell T. Fully convolutional networks for semantic segmentation. Proceedings of the IEEE/CVF Conference on Computer Vision and Pattern Recognition.

[48] Ronneberger O., Fischer P., Brox T. U-Net: Convolutional networks for biomedical image segmentation. Proceedings of the International Conference on Medical Image Computing and Computer-Assisted Intervention.

[49] Badrinarayanan V., Kendall A., Cipolla R. (2017). Segnet: A deep convolutional encoder-decoder architecture for image segmentation. IEEE Trans. Pattern Anal. Mach. Intell..

[50] Chen L.-C., Papandreou G., Kokkinos I., Murphy K., Yuille A.L. (2017). Deeplab: Semantic image segmentation with deep convolutional nets, atrous convolution, and fully connected CRFs. IEEE Trans. Pattern Anal. Mach. Intell..

[51] Park S.J., Hong K.S., Lee S. Rdfnet: Rgb-d multi-level residual feature fusion for indoor semantic segmentation. Proceedings of the IEEE International Conference on Computer Vision.

[52] Zhang Z.Y. (2000). A flexible new technique for camera calibration. IEEE Trans. Pattern Anal. Mach. Intell..

[53] Li H.S., Cao Z.J. (2016). Matlab codes of subset simulation for reliability analysis and structural optimization. Struct. Multidisc. Optim..

[54] Xu H., Zhang J. AANet: Adaptive aggregation network for efficient stereo matching. Proceedings of the IEEE/CVF Conference on Computer Vision and Pattern Recognition.

[55] Gu X., Fan Z., Zhu S., Dai Z., Tan F., Tan P. Cascade cost volume for high-resolution multi-view stereo and stereo matching. Proceedings of the IEEE/CVF Conference on Computer Vision and Pattern Recognition.

[56] Ilg E., Mayer N., Saikia T., Keuper M., Dosovitskiy A., Brox T. Flownet 2.0: Evolution of optical flow estimation with deep networks. Proceedings of the IEEE/CVF Conference on Computer Vision and Pattern Recognition.

[57] Chabra R., Straub J., Sweeney C., Newcombe R., Fuchs H. Stereodrnet: Dilated residual stereonet. Proceedings of the IEEE/CVF Conference on Computer Vision and Pattern Recognition.

[58] Kingma D.P., Ba J. (2014). Adam: A method for stochastic optimization. arXiv.

[59] Jiang J., Zheng L., Luo F., Zhang Z. (2018). Rednet: Residual encoder-decoder network for indoor rgb-d semantic segmentation. arXiv.

[60] Smith P.R. (1981). Bilinear interpolation of digital images. Ultramicroscopy.

[61] Li H., Ding W., Cao X., Liu C. (2017). Image registration and fusion of visible and infrared integrated camera for medium-altitude unmanned aerial vehicle remote sensing. Remote Sens..

[62] Torr P.H.S., Murray D.W. (1997). The development and comparison of robust methods for estimating the fundamental matrix. Int. J. Comput. Vis..

[63] Seshadri V., Hsieh K., Boroum A., Lee D., Kozuch M.A., Mutlu O., Gibbons P.B., Mowry T.C. (2015). Fast bulk bitwise AND and OR in DRAM. IEEE Comput. Archit. Lett..

[64] Otsu N. (1979). A threshold selection method from gray-level histograms. IEEE Trans. Syst. Man. Cybern..

[65] Patel S., Ramzan Z., Sundaram G.S. Luby-Racko. Ciphers: Why XOR is not so exclusive. Proceedings of the International Workshop on Selected Areas in Cryptography.

[66] Dziubich T., Szymanski J., Brzeski A., Cychnerski J., Korlub W. (2016). Depth images filtering in distributed streaming. Polish Marit. Res..

[67] Bloch V., Barchilon N., Halachmi I., Druyan S. (2020). Automatic broiler temperature measuring by thermal camera. Biosyst. Eng..

[68] Hirschmueller H. (2008). Stereo processing by semiglobal matching and mutual information. IEEE Trans. Pattern Anal. Mach. Intell..

[69] Mei X., Sun X., Zhou M., Jiao S., Wang H., Zhang X. On building an accurate stereo matching system on graphics hardware. Proceedings of the IEEE International Conference on Computer Vision Workshops.

[70] Bleyer M., Rhemann C., Rother C. PatchMatch stereo-stereo matching with slanted support windows. Proceedings of the British Machine Vision Conference.

[71] Russell B.C., Torralba A., Murphy K.P., Freeman W.T. (2008). LabelMe: A database and web-based tool for image annotation. Int. J. Comput. Vis..

[72] Guo Y., Aggrey S.E., Oladeinde A., Johnson J., Zock G., Chai L. (2021). A machine vision-based method optimized for restoring broiler chicken images occluded by feeding and drinking equipment. Animals.

[73] Veeramani B., Raymond J.W., Chanda P. (2018). DeepSort: Deep convolutional networks for sorting haploid maize seeds. BMC Bioinformatics.

